# Nutrition and functional compounds of traditional foods in migrant women with schizophrenia: A systematic review

**DOI:** 10.1192/j.eurpsy.2025.2407

**Published:** 2025-08-26

**Authors:** A. González- Rodríguez, M. Natividad, R. Penadés, N. Bagué, A. Balagué, J. P. Paolini San Miguel, E. Izquierdo, M. Salvador, A. Vallet, A. Pérez, J. A. Monreal

**Affiliations:** 1 Mental Health, Mutua Terrassa University Hospital. University of Barcelona. CIBERSAM; 2 Mental Health, Mutua Terrassa University Hospital. University of Barcelona, Terrassa; 3 Psychiatry and Psychology, Barcelona Clinic Schizophrenia Unit (BCSU). Hospital Clinic of Barcelona. IDIBAPS- CIBERSAM., Barcelona, Spain

## Abstract

**Introduction:**

Several studies have reported that high fruit and vegetable consumption is associated with increased life expectancy. In migrant populations, lifestyle habits are modified in the process of acculturation.

**Objectives:**

Our aim was to review the diversity/functional compounds of food groups in Latin American, African, Asian and European cultures. In a second step, we aimed to review the dietary patterns for migrant women with schizophrenia.

**Methods:**

A two-stage systematic review was conducted using the PubMed and ClinicalTrials.gov databases (2004-2024). The first part included studies reporting information on food and nutrients in adult populations from the nationalities with the highest prevalence of women with schizophrenia attending the Mútua Terrassa Functional Unit for Women with Schizophrenia (Dominican Republic, Venezuela, Ecuador, Morocco, Senegal, Romania and Pakistan). In a second part papers focused on food consumption among migrant women with schizophrenia.

**Results:**

A total of 87 studies were included from a total of 21,306 records screened. First part: (1) Latin America (n=32). Outcomes:food choice trajectories for dietary acculturation, barriers and facilitators for fruit and vegetable consumption (antioxidant effects of polyphenols), fruit/tubers/legumes traditionally consumed. (2) Africa (n=25). Regular consumption of oils, changes in medications during the holy month of Ramadan, anti-inflammatory effects of species. (3) Europe (n=17). Antioxidant properties of phenolics in mushrooms, and polyphenols in berries. (4) Asia (n=13). Women more vegetables, fruit and fish. Second part: Dietary intake of vitamin C, niacin, and folate reduced in schizophrenia. Few studies in women with schizophrenia.

**Conclusions:**

Nutritional intervention programmes for migrant women with schizophrenia should pay attention to biocultural heritage and traditional antioxidant/anti-inflammatory foods.

**Disclosure of Interest:**

None Declared

